# The Secrets of Economy in Institutional Housekeeping

**Published:** 1913-11-15

**Authors:** 


### INSTITUTIONAL HOUSEKEEPING.

### The Secrets of Economy.

Rightly to envisage the subject of the commissariat it is necessary first to decide upon the ratio of expenditure which is suitable for the persons who are to be boarded. The tune must be set before the household can march in time to it. In workhouses the inmates usually cost about 4s. a week per head. Patients on milk diet cost about 3s. 6d. a week. Good diet for persons in ordinary health and full work costs from 6s. to 10s. a week, according to the amount of variety and non-essential elements introduced into it. No good housekeeper can do her best unless she be given to understand at what rate she is expected to cater for the household, and to expect her " to be as economical as possible " without specifying the sum which she is to spend is as unreasonable as to expect an orchestra to keep time without the aid of the conductor's baton.

### The Hospital Problem.

In every hospital the ratio of expenditure on the commissariat must vary according to the class of persons to be fed. In an examination made some years ago into the cost of boarding different sections of the household in a well-managed hospital it was ascertained that while the total cost of all the provisions divided among all the persons boarded in the establishment worked out at 10d. per day per head, this amount was not the cost of boarding any one of them. At the hospital in question the patients' board cost 8½d. a day; that of the nurses cost 1s. 3½d. a day; that of the servants cost 8½d. a day; and the medical officers' board, including wine, cost 3s. a day.* With the admirable system of book-keeping in use the actual expenditure on every individual was ascertainable. But in hospitals which make no endeavour to separate the various strands of their household all is uncertain; efforts at economy too often take the form of stinting in common necessaries, while everything nice is tabooed as extravagant. Yet in such institutions waste may be permitted in the kitchen which would suffice to board an extra ten per cent. of inmates.

### How to Separate the Strands.

In very large establishments medical officers, nurses, domestics, and patients are boarded as we have shown, at widely differing rates. But in medium-sized hospitals it is found in practice that the cost of boarding the servants is very little less than that of boarding the nurses, and that the medical officers cost very little more, receiving much the same fare as the nursing staff. The problem in the large establishment is to separate the cost of the commissariat into four strands, that of the small hospital is to separate it into two.

A system which groups together all the able-bodied men and women on the one hand and provides them with generous board is in our judgment to be preferred for institution purposes to that which marks out a special high table for one group of officers, provided without regard for economy. But how can the cost of the staff dietary be separated from that of the patients fed under the same roof? How does the housekeeper distinguish between the provisions sent in by the same tradesmen and cooked in the same kitchen?

### No Separate Kitchen Necessary.

The simplest way of differentiating the cost of the nurses' and officers' board from that of the patients is to have a separate kitchen and cook for the nurses' home, while the officers' board is contracted for or supplied by the college. But this plan is not a complete panacea for entangled accounts. It will be found in practice that even in hospitals provided with separate kitchens a certain amount of food for others besides patients has to be cooked in the hospital kitchen, and without a good system of accounts the cost of each section of the household will still be unascertainable. On the other hand, by means of a good system it is possible to keep entirely distinct the cost of boarding two, three, or four sections of the household whose food is all prepared under one cook, in one kitchen. How is it done? At St. Bartholomew's Hospital the kitchen serves the needs of close on a thousand persons. At the head of this well-organised department is the head cook, and under her are nine kitchenmaids. The preparation of the food for the various sections of the household is divided among various maids, each responsible for her own department. At one side, for instance, is the place appointed for the maid whose sole duty is the patients' fish. Another maid has charge of provisions for the nurses' table. At another pastry and sweets are made for the officers' table. Another maid has charge of the rations for the domestic staff. All supplies, as requisitioned by the housekeeper, are referred to their proper place, and no confusion can take place because no overlapping is ever allowed. Convalescent patients, nursing staff, medical officers, and domestics may all have roast beef for dinner, yet the joints for each table are kept entirely distinct, following the requisition sheet made out the day before. Any meat or other kind of food left over is returned to its own department, and through all the immense coming and going of provisions a clearly marked dividing line of administration keeps the food for each section of the household distinct no less definitely than if four separate kitchens were at work. The same admirable organisation may be seen at work in the big kitchen at St. George's Hospital, though there the numbers are far less. So far, then, as regards kitchen management no difficulty need be anticipated in keeping the food supplies distinct. All that is needed is a cook trained to observe a good system and capable of training her subordinates.

* See *The Matron*, p. 55. (The Scientific Press, 28 and 29 Southampton Street, Strand, London, W.C. Price 2s. 6d.)

### Good Book-keeping Essential.

Although the crux of good housekeeping lies in the kitchen, yet it is also true that the cook can do little as regards separating expenditure unless the establishment be directed by a sensible system of accounts. To order for each section of the household the exact quantity of every commodity needed, and to enter under its proper heading the cost of whatever is consumed, is the problem which confronts the steward or housekeeper as the case may be. So soon as an attempt is made to organise the commissariat into separate strands it becomes apparent that some articles of the daily rations fall under a different category to others. Certain articles of diet pass straight to the kitchen to be cooked. Other articles, such as milk, lump sugar, butter, jam, etc., need not go to the kitchen at all. In addition to this division of all articles into cooked and uncooked there is a further distinction to be observed.

Certain articles arrive from the tradesmen or contractors under a daily order, such as meat, bread, vegetables, etc. Other articles must necessarily be kept in stock, even though the quantity stored be kept as low as possible. Such articles are sugars, farinaceous foods, tea, coffee, etc. Accordingly, when the requisitions are made out provisions which need cooking will follow a different course to those which are not to be cooked, and provisions kept in stock must be requisitioned separately from those supplied daily from outside. To organise the supplies so that these distinctions are not permitted to introduce any element of uncertainty into the accounts is the first step towards complete control over the commissariat.

### The Combined Diet-Sheet.

In most well-managed hospitals the plan adopted is somewhat as follows: Early in the afternoon the housekeeper receives the diet-sheets for the following day from each ward, and combines them into a sheet which shows the quantities of each article required for the use of all the patients. She then makes her own estimates of what is required for the nurses', officers', and domestics' tables, either preparing the menus herself or receiving them from the matron or other authority. Having estimated to the best of her ability, in consultation with the head cook and after inspection of the larders, the exact amount of every article of food required during the next four-and-twenty hours, the sheets containing the requisitions are passed on to the steward, and the task of combining them is perfectly simple.

### The St. George's Hospital System.

At St. George's Hospital the steward orders all the articles which do not require cooking, supplying articles kept in stock from the store-rooms. Provisions which need cooking are requisitioned by the housekeeper. This is merely a matter of administrative detail. But whatever method of ordering supplies be adopted, the classified diet-sheet, stating the quantities of each article consumed by each section of the household, is the crux of the system. When this is once in use the problem of ascertaining the average cost of each section of the household is reduced to a mere question of clerical work. The price of every article is known. A junior clerk can work out the cost once a week in a few hours.

It is essential to accurate knowledge of the rate at which each individual is boarded that the numbers who dine every day be entered in a book kept for that purpose by the housekeeper. Numbers inevitably vary a good deal, and it is the greatest possible service to cook and housekeeper to know the rate per head per week at which the cost of the food works out. We append an abstract of the sheet in use at St. George's Hospital for setting out the quantities of each article of diet used daily and weekly by officers, patients, nurses, and household.

Housekeeper's Daily Requisition Sheet.

| — | Patients | Medical Officers | Nurses | House |
|---|---|---|---|---|
| Meat: | | | | |
| 1. Beef | | | | |
| 2. Mutton | | | | |
| 3. Shin for beef-tea, etc. | | | | |
| Fish: | | | | |
| 1. Ordinary diets | | | | |
| 2. Soles, etc. | | | | |
| Poultry: | | | | |
| 1. Fowls | | | | |
| 2. Rabbits | | | | |
| Bread: | | | | |
| 1. Bread | | | | |
| 2. Flour | | | | |
| 3. Cakes, etc. | | | | |
| Vegetables and Fruit: | | | | |
| 1. Potatoes | | | | |
| 2. Other Vegetables | | | | |
| 3. Fruit | | | | |
| Milk: | | | | |
| 1. Milk | | | | |
| 2. Cream | | | | |
| Cheesemongery: | | | | |
| 1. Butter | | | | |
| 2. Cheese | | | | |
| 3. Bacon, etc. | | | | |
| Eggs: | | | | |
| 1. Cooking | | | | |
| 2. New laid | | | | |
| Grocery: | | | | |
| 1. Tea | | | | |
| 2. Coffee | | | | |
| 3. Jams, etc., etc. | | | | |
| Aerated Waters | | | | |
| Alcohol: Various headings | | | | |

| — | Officers | | | Patients | | | Notes | Nursing Staff—Ward Maids | | | Servants | | |
|---|---|---|---|---|---|---|---|---|---|---|---|---|---|
| | Sunday | Mon., etc. | Total | Sunday | Mon., etc. | Total | | Sunday | Mon., etc. | Total | Sunday | Mon., etc. | Total |
| Bacon | | | | | | | | | | | | | |
| Baking Powder | | | | | | | | | | | | | |
| Barley, Pearl | | | | | | | | | | | | | |
| Bovril | | | | | | | | | | | | | |
| Brawn | | | | | | | | | | | | | |
| Bread | | | | | | | | | | | | | |
| Butter, Fresh | | | | | | | | | | | | | |
| ,, Cooking | | | | | | | | | | | | | |
| ,, Dorset | | | | | | | | | | | | | |
| Suet | | | | | | | | | | | | | |
| Sugar, L. | | | | | | | | | | | | | |
| ,, M. | | | | | | | | | | | | | |
| ,, C. | | | | | | | | | | | | | |
| Sultanas | | | | | | | | | | | | | |
| Syrup, Golden (½-cwt. drums) | | | | | | | | | | | | | |
| Tea | | | | | | | | | | | | | |
| Vegetables... | | | | | | | | | | | | | |
| Vermicelli | | | | | | | | | | | | | |
| Vinegar | | | | | | | | | | | | | |
| etc., etc. | | | | | | | | | | | | | |

